# In vitro characterization of Bacillus sp. LS100 for detoxifying deoxynivalenol

**DOI:** 10.1099/mic.0.001730

**Published:** 2026-10-15

**Authors:** Changning Yu, Hai Yu, Tracy Guo, Honghui Zhu, Lili Mats, Chengbo Yang, Qi Wang, Joshua Gong

**Affiliations:** 1 Department of Animal Science, University of Manitoba, Winnipeg, Manitoba, R3T 2N2, Canada; 2 Guelph Research and Development Centre, Agriculture and Agri-Food Canada, Guelph, Ontario, N1G 5C9, Canada

**Keywords:** *Bacillus *sp. LS100, deoxynivalenol, detoxification, encapsulation, stability

## Abstract

The mycotoxin deoxynivalenol (DON) contamination in animal feed has been reported to reduce the growth performance of animals and compromise their immune system, especially pigs. Bacterial detoxification is considered a promising strategy to mitigate its harmful effects. We previously identified a bacterial isolate (*Bacillus* sp. LS100) from the chicken gut with the ability to detoxify DON. To explore its potential application in swine production, the current study was focused on *in vitro* characterization of *Bacillus* sp. LS100 with regard to its capacity to detoxify DON. The bacterium was stable in keeping its activity to fully detoxify DON when subcultured for 12 passages under anaerobic conditions. The bacterial culture was successfully scaled up and stored for at least 1 month without losing any activity to detoxify DON. In addition, it was able to fully detoxify DON in mouldy corn with a diluted medium in an anaerobic atmosphere. While the bacterium exhibited a certain degree of tolerance to oxygen, it was sensitive to low pH and bile salts, including pig gastric fluid. In the presence of digesta from the pig jejunum or ileum, the bacteria retained near-full or full detoxification activity of DON after 24 or 48 h incubation under anaerobic conditions. To protect *Bacillus* sp. LS100 from the detrimental effects of gastric acid and bile salt, the bacterium was encapsulated anaerobically. The encapsulated bacteria stored in wet beads at 22 °C exhibited greater stability and retained detoxification activity longer than those stored in dry beads. After exposure to simulated gastric fluid for 2 h, the bacterium in the wet beads retained the DON detoxification activity. The current study suggests that, when adequately protected, *Bacillus* sp. LS100 holds promise as a viable strategy for DON removal in the pig gut.

## Data Availability

All data generated from the current study are included in this article. The datasets used and/or analysed during the study are available from the corresponding author upon request.

## Key points

1. *Bacillus* sp. LS100 grew and detoxified deoxynivalenol (DON) under the conditions mimicking the gut environment, except for low pH and high bile salts.
2. The scaled-up bacterial culture retained the function to detoxify DON after 1 month of storage at 22 °C.
3. Microencapsulation protected the bacterium and its detoxification activity.

## Introduction

The predominant mycotoxins in cereal samples in North America are deoxynivalenol (DON), fumonisins and zearalenone, with DON exhibiting the highest prevalence at up to 64.1% [1]. DON is a common mycotoxin contaminant in cereal grains, primarily produced by *Fusarium* fungi. It is highly stable under heat, pressure and acidic conditions, making it more likely to persist and occur in food or animal feed than other mycotoxins [2, 3]. Pigs, especially piglets, are the most sensitive to DON exposure amongst food-producing animals [4, 5]. Ingestion of DON-contaminated feed may result in a spectrum of adverse effects in pigs, including reduced appetite, diarrhoea, vomiting, impaired feed efficiency, weight loss, immunosuppression and, in severe cases, mortality [6, 7]. To mitigate such risks, the Canadian Food Inspection Agency has established a guidance value of 1.0 mg kg^−1^ (p.p.m.) as the maximum allowable DON concentration in the daily pig ration. However, it is noteworthy that the values at, or even below, this threshold can still negatively affect pig health. For example, Jia *et al*. [8] reported that adding a low dose of DON (1.06 mg kg^−1^) to piglet diets led to heightened regulation of pro-inflammatory cytokines in the jejunum, along with alterations in gut microbiota composition, despite no discernible impacts on growth performance.

Given the widespread occurrence of DON in cereals, detoxifying this mycotoxin represents a significant challenge. While physical and chemical methods can offer partial mitigation of DON contamination, their inherent limitations, including a lack of selectivity and adverse effects on the nutritional quality of cereals, render them inadequate as comprehensive solutions [9]. Utilizing biological detoxification methods involving micro-organisms and enzymes can serve as an efficacious strategy to overcome the constraints associated with physical and chemical approaches in DON management. In recent years, extensive research has been conducted on various micro-organisms and enzymes for DON detoxification [10]. Nevertheless, to date, the European Commission has approved only *Eubacterium* stain BBSH 797 as a commercial feed additive for DON or zearalenone detoxification [11]. There is an urgent need for the development of additional micro-organisms or enzymes capable of detoxifying DON for use in animal feed. A bacterial isolate (named *Bacillus* sp. LS100) was selected from chicken gut digesta, which could completely convert 100 µg ml^−1^ DON into the less toxic compound de-epoxy deoxynivalenol (DOM-1) within a 72 h incubation at 37 °C under anaerobic conditions [12]. This isolate exhibited consistent and high detoxification efficacy across ten consecutive sub-cultures. When incubated at 37 °C for 72 h at 4×10^9^ c.f.u. ml^−1^, the isolate successfully converted DON (7.6 mg kg^−1^) to DOM-1 (4.3 mg kg^−1^) in mouldy corn through a pretreatment process [13]. In addition, incorporating such pretreated mouldy corn containing the mixture of DOM-1 and metabolites of the isolate in the diets did not lead to statistically significant alterations in nutrient composition, feed palatability and pig growth performance. Although this isolate has been patented [14, 15], further *in vitro* characterization is required to validate its stability and functional reliability under the conditions mimicking the pig gastrointestinal environment prior to the industry-scale application in swine. Therefore, the aim of the current study was to further our previous studies and investigate the stability of the bacterium and its detoxification activity of DON in the presence of oxygen, low pH, high bile salts and gastric and digestive fluids. To protect the bacterium and maintain its activity under such conditions, encapsulation of the bacterial cells was also explored, pointing to a potential path forward for its application in swine production.

## Methods

### Bacterial culturing and detoxification analysis

The bacterial isolate *Bacillus* sp. LS100 from the chicken gut with the ability to detoxify DON was reported previously, including the use of chickens for research that complied with the University of Guelph Animal Care Committee Guidelines [12]. All the bacterial culturing and subculturing in different experiments were conducted in L10 broth (unless indicated otherwise) at 37 °C in an anaerobic atmosphere composed of 5% H_2_, 10% CO_2_ and 85% N_2_ (COY Laboratory Products Inc., Grass Lake, MI, USA), as described previously [12]. The inoculum was 10% for all the bacterial cultures. To examine if the bacterium requires the presence of DON during sequential subculturing to retain its detoxification activity, two procedures were used. First, DON (25 p.p.m.) was included in L10 broth for subculturing from the first to twelfth passages. In parallel, the second procedure included the subculturing from the first to ninth passages in the absence of DON, followed by the tenth to twelfth passages in the presence of 25 p.p.m. DON.

For the detoxification activity assay, 25 p.p.m. of DON was included in L10 broth (unless indicated otherwise) and incubated with the bacterium for 48 h anaerobically. The detoxification from DON to DOM-1 was evaluated by either liquid chromatography–MS (LC-MS) using the method described by Young *et al*. [16] with some modifications, or HPLC analysis as reported previously [13, 17]. The HPLC analysis was used to determine the conversion of DON to DOM-1 by *Bacillus* sp. LS100 in the experiment of encapsulating the bacterium, while the LC-MS analysis was used to measure the conversion in the rest of the experiments. The detailed key information of LC-MS/MS analysis after the modifications included the use of a Thermo^®^ Scientific Q-Exactive^™^ Orbitrap mass spectrometer, which was equipped with a Vanquish^™^ Flex Binary UPLC System (Waltham, MA, USA) and used an Eclipse Plus C18 column (100×2.1 mm, 3.5 µm, Agilent, Santa Clara, CA, USA). The binary mobile phases consisted of solvent A (99.9% H_2_O/0.1% formic acid) and solvent B (99.9% MeOH/0.1% formic acid). The used solvent gradient was composed of 10–55% B for 0–5 min, 55–100% B for 5–6 min, 100–100% B for 6–8 min, 100–10% B for 8–9 min and 10% B for 9–15 min. The column temperature was set at 25 °C with the flow rate at 0.4 ml min^−1^ and the injection volume of 2 µl. ESI positive ionization mode was used, and the spray voltage was set at 4.5 kV. MS data were collected using the Full-MS/DDMS2 (TopN=10) method, and the data were visualized and analysed using Thermo FreeStyle^™^ 1.7PS2 software. Under these conditions, the limit of detection (LOD) and limit of quantification (LOQ) of DON were determined to be 0.131 and 0.397 p.p.m., respectively.

The samples of stomach fluid (SF), jejunal digesta (JED) and ileal digesta (ILD) used for the analysis of DON detoxification were collected from adult pigs (100 days old). The animal experiment and all measurements were approved by the Animal Care Committee of the University of Manitoba (AC#F21-002). The pigs were handled according to the guidelines described by the Canadian Council on Animal Care (CCAC, 2009 - Guidelines on: the care and use of farm animals in research; Teach. Test. II. 103–125). All the microbiological experiments in the present study were performed in an anaerobic atmosphere, except for the experiment to examine the tolerance of *Bacillus* sp. LS100 to oxygen, the samples serving as the control in the culture scale-up test and the storage of encapsulated *Bacillus* sp. LS100 in the form of dry beads. A large volume of buffers, solutions or media was prepared by heating and flushing with nitrogen or mixed gas (5% H_2_, 10% CO_2_ and 85% N_2_), while a small volume of buffers or solutions was equilibrated inside the anaerobic chamber for 24–48 h to remove oxygen. All the assays were conducted in triplicate, and the results are presented in the average value±standard deviations.

### Sensitivity test of the bacterial growth at low pH or high bile salts

The sensitivity in growth of *Bacillus* sp. LS100 to low pH was evaluated using tryptic soy broth (TSB) with different pH values (2.0, 3.0, 5.0 and 7.2). Concentrated hydrochloric acid (HCl) was added to 20 ml of TSB to achieve the desired pH values (±0.05). Subsequently, 0.2 ml of *Bacillus* sp. LS100 was mixed with 1.8 ml of the pH-adjusted TSB. Following 24 h of anaerobic incubation, the optical density of the cultures was measured at 600 nm.

The TSB was also used to evaluate the sensitivity of *Bacillus* sp. LS100 in growth to bile salts with different supplementation levels of the salts from 0.1% up to 1.0% (w/v). Two different inoculum levels (1% or 10%) of *Bacillus* sp. LS100 were tested. After 24 h of anaerobic incubation, the optical density of the cultures was measured at 600 nm.

### Oxygen tolerance test

To determine the tolerance of *Bacillus* sp. LS100 to oxygen with regard to its detoxification activity, an anaerobically cultivated culture (5 ml in a 12 ml culture tube; incubated at 37 °C for 48 h) was exposed to oxygen by incubation on the lab bench outside the anaerobic chamber at 22 °C without agitation, with the tube lid covered (left loose), for a duration of 22 days. Within the period of 22 days, the culture was monitored for its detoxification activity by sampling once every 2 days for the first 11 days, followed by a detoxification assay inside the anaerobic chamber through a subculture to determine the effect of exposure to oxygen on the detoxification activity of *Bacillus* sp. LS100. Each time before the sampling, the bacterial culture on the bench was vortexed until it became pink, indicating the existence of oxygen. The sampled culture was then brought back to the anaerobic chamber, and 0.2 ml of the sampled culture was mixed with 1.8 ml of L10 broth containing 25 p.p.m. of DON. After 48 h incubation under anaerobic conditions at 37 °C, the conversion of DON to DOM-1 by the subculture was then determined.

### Culture scale-up and storage test

To examine the effect of culture scale-up and storage of *Bacillus* sp. LS100 on its activity to detoxify DON, 30 ml of the 48 h-grown bacterium was inoculated into 300 ml of brain heart infusion (BHI) broth in a 500 ml serum bottle followed by 48 h incubation at 37 °C without agitation. After three passages of subculturing, the 48 h-grown culture was stored at 22 °C for up to 44 days and was then examined for the activity of DON breakdown at different time intervals. All the cultures, including the initial inoculum and subcultures in BHI, sampling and storage, were kept anaerobically. In parallel, the same procedure was carried out aerobically with a 500 ml serum bottle (including the use of BHI without removing oxygen) to serve as a control, except for the initial inoculum that was grown anaerobically for 48 h.

### Test for detoxification activity in the presence of pig SF or gut digesta

To evaluate the ability of *Bacillus* sp. LS100 to detoxify DON in the presence of pig SF, 0.5 ml of the bacterial culture in L10 broth was first harvested and resuspended in either pig SF for 1, 2 or 3 h or in PBS for 3 h as a control. Following the treatment, SF or PBS was removed, and the bacterial pellet was resuspended in 0.5 ml of L10 broth. A total of 0.2 ml of this suspension was then mixed with 1.8 ml of L10 broth containing 25 p.p.m. DON. Bacterial growth and DON detoxification activity were assessed after 24 and 48 h of anaerobic incubation, respectively.

To examine the ability of *Bacillus* sp. LS100 to detoxify DON in the presence of pig digesta, 0.2 g of either ileal or JED from pigs was added to an assay mixture containing 1.8 ml of PBS, L10 broth or 0.9 ml each of PBS and L10 broth, which had 0.2 ml of the bacterial inoculum and 25 p.p.m. DON. DON conversion to DOM-1 was determined after 12, 24 and 48 h of anaerobic incubation.

### Mouldy corn test

*Fusarium*-infected corn was prepared and harvested as described previously [13]. Mouldy kernels collected from the infected ears were ground, passed through a 10-mesh screen and then autoclaved at 121 °C for 15 min. The resulting ground mouldy corn was analysed, and the DON concentration was 124 µg g^−1^. To examine the ability of *Bacillus* sp. LS100 to detoxify DON in the mouldy corn in either L10 or TSB broth diluted with PBS (pH 7.2), the assay mixture (2.0 ml), which was composed of 0.1 g mouldy corn, 0.2 ml bacterial inoculum and 1.8 ml broth containing different volumes of PBS at the final concentrations of 30, 50, 70 or 80%, was incubated at 37 °C for 24 and 48 h anaerobically followed by a detoxification activity assay.

### Encapsulation of *Bacillus* sp. LS100 and its storage test

To preserve its detoxification activity, *Bacillus* sp. LS100 cells were encapsulated in alginate-based microbeads under anaerobic conditions. Briefly, the bacterial cells were harvested by centrifugation from a fully grown culture and resuspended in PBS to a final concentration of 10^9^ cells ml^−1^. This cell suspension was mixed with an alginate–whey protein solution containing 2.0% (w/v) alginate and 0.6% (w/v) whey protein at a 1 : 9 ratio. The mixture was then manually extruded through a syringe needle (22 G in size) into a 1.0% (w/v) calcium chloride solution. The resulting microbeads were kept stirring in the calcium chloride solution for 30 min before being collected by filtration. The microbeads were then equally divided into two portions: one portion was immersed in PBS and sealed in a bottle (as wet beads) prior to the transfer outside the anaerobic chamber and storage at 22 °C; the other was placed in an open bottle at 22 °C to air-dry outside the anaerobic chamber for 24 h (as dry beads), followed by storage at 22 °C in a sealed bottle. To assess DON-detoxification activity, the encapsulated *Bacillus* sp. LS100, in either wet or dry beads, was returned to the anaerobic chamber, resuspended in simulated intestinal fluid (pH 7.2) to un-encapsulate the bacterium and then incubated in L10 broth containing 10 p.p.m. DON after 8, 10 and 14 days of storage.

The wet beads stored for 14 days were also examined for the detoxification activity after incubation in simulated gastric fluid (SGF, pH 2.0) for 2 h. The detoxification activity assay was conducted inside the anaerobic chamber through the procedure described above. Unencapsulated *Bacillus* sp. LS100 was treated in parallel to serve as a control.

### Data analysis

The DON conversion data were analysed using PROC GLM followed by Tukey’s multiple comparison test (SAS 9.4). A *P*-value of 0.05 was chosen to indicate significance in difference.

## Results and discussion

### Bacterial growth and its detoxification activity

Fig. 1 shows the relationship of the growth and DON removal activity of *Bacillus* sp. LS100 in L10 broth. The bacterium did not demonstrate a full activity until its cell density reached 10^9^ c.f.u. ml^−1^, suggesting a prerequisite of bacterial full growth for the activity.

**Fig. 1.**
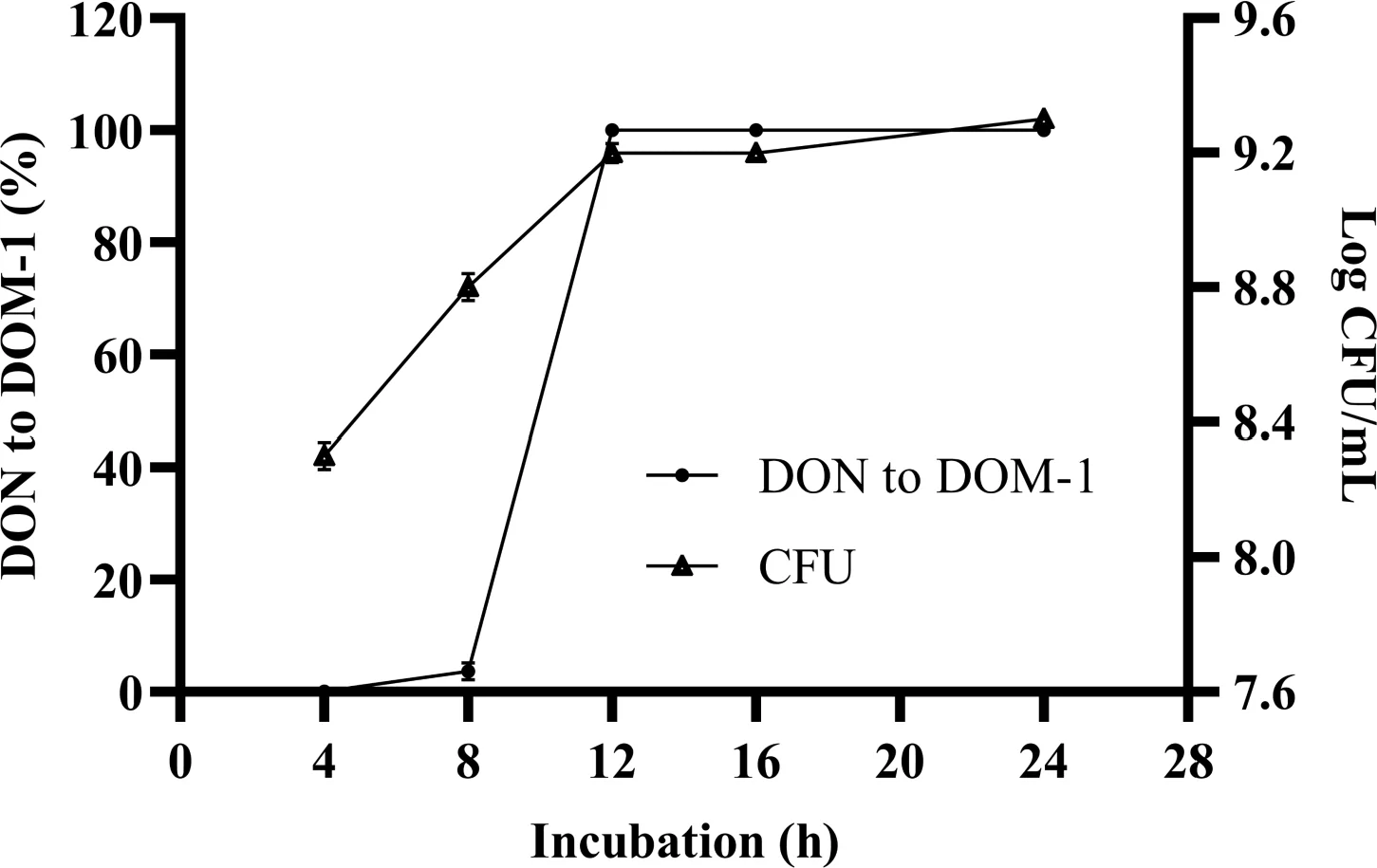
Relationship of the growth and DON detoxification activity of *Bacillus* sp. LS100 in L10 broth. *N*=3.

### Effect of media on detoxification activity

Table 1 displays the detoxification activity of *Bacillus* sp. LS100 incubated in L10, TSB or BHI broth for 24 and 48 h anaerobically. The bacterium fully detoxified DON in both L10 and BHI, but not in TSB, after 24 h incubation, although its cell density in all three broths reached 10^9^ c.f.u. ml^−1^. After 48 h incubation, DON in all the tested broths was fully converted to DOM-1. These results indicate that both L10 and BHI better support the detoxification activity of the bacterium than TSB. Previously, we examined seven different media for their effects on the growth of the bacterium and its DON detoxification activity [12]. Only L10 and AIM (anaerobic incubation medium)+CecExt (caecal content extract) media fully supported the transformation of DON to DOM-1. While the bacterial cultures could be rapidly established in L10 broth, the growth of bacteria in AIM+CecExt was minimal. In the present study, both BHI and TSB could enable the rapid growth of the bacterium. In addition, compared with L10 and AIM+CecExt, which each contain multiple components and have to be made manually, both BHI and TSB are commercially available, which makes research and future application of the bacterium more efficient and cost-effective.

**Table 1.** Percent of DON removed by *Bacillus* sp. LS100 grown in L10, TSB or BHI broth for 24 and 48 h

Medium	Incubation (h)	
	24	48
L10	100.0±0.00^a^	100.0±0.00
TSB	30.57±12.30^b^	100.0±0.00
BHI	100.0±0.00^a^	100.0±0.00

Note: When the DON level in the assay was below LOD (0.131 p.p.m.) or LOQ (0.397 p.p.m.), DON breakdown (DON to DOM-1) was regarded as 100%. This is applied to other tables and figures in the present study. *N*=3. The values labelled with different letters in a column are significantly different (*P*≤0.05).

### DON-detoxifying activity during subcultures

The stability of a bacterium’s detoxification activity during subculturing is a critical parameter for its potential commercial application. In the present study, subculturing of *Bacillus* sp. LS100 in the presence and absence of DON was examined. As shown in Table 2, the bacterium consistently maintained its ability to detoxify DON under anaerobic conditions through at least 12 successive subcultures in L10 broth. All subcultures (from the first to the twelfth) retained full activity in converting DON to DOM-1, even when the initial nine subcultures were propagated in the absence of DON, provided that they reached full growth under anaerobic conditions. These results also suggest that DON is not required in the broth for the bacterium to keep its detoxification activity during subculturing. Prior to our observation, He *et al*. [18] reported that micro-organisms as a mixed population in the digesta from the chicken large intestine could retain the activity to detoxify 98% of DON by the sixth passage of subculturing. However, no detoxification of DON was detected by the second passage of the micro-organisms from soil, the pig large intestine or rumen fluid during subculturing. Recently, Wu *et al*. [19] documented that a bacterial consortium subculture GMmixP3, isolated from the intestines of free-range chickens, was capable of completely degrading DON at 37 °C under anaerobic conditions within 5 days. However, the degradation rate markedly decreased to 31.46% under aerobic conditions. Similar to the results from the present study, Wang *et al*. [20] showed that a bacterial consortium IFSN-C1, isolated from wheat leaves in Jiangsu Province, China, increased in abundance during the first 7 subcultures and retained its DON-degrading ability after 50 subcultures with a degradation rate over 80%. These previous reports suggest that bacterial isolates or populations from different sources can vary significantly in their ability to detoxify DON during subculturing. The present finding that *Bacillus* sp. LS100 preserved full DON detoxification capability through the twelfth passage of subculturing highlights its potential as a robust microbial candidate for use as a feed additive in mitigating DON contamination.

**Table 2.** Stability of anaerobically grown subcultures of *Bacillus* sp. LS100 in detoxifying DON

Subculture	DON to DOM-1 (%)	
	With DON	Without DON
S-1	100	UAT
S-2	100	UAT
S-3	100	UAT
S-4	100	UAT
S-5	100	UAT
S-6	100	UAT
S-7	100	UAT
S-8	100	UAT
S-9	100	UAT
S-10	100	100
S-11	100	100
S-12	100	100

Note: The bacterium was sub-cultured in L10 broth. UAT: unable to test the activity since the substrate (DON) was not included in the broth. *N*=3.

### Tolerance to oxygen

To facilitate industrial applications, the oxygen tolerance of anaerobic bacteria is a critical factor. As shown in Fig. 2, when the full-growth culture (initial culture grown inside the anaerobic chamber) of *Bacillus* sp. LS100 in L10 broth was kept outside the anaerobic chamber at 22 °C for 22 days, the initial culture maintained its detoxification activity for up to 11 days upon being returned to anaerobic conditions for the detoxification assay. During each sampling within this period, the initial culture became pink, indicating that the culture was not oxygen-free. These data suggest that *Bacillus* sp. LS100 possesses a certain degree of oxygen tolerance. Specifically, the bacterium appeared to be oxygen-tolerant for growth but required anaerobic conditions (low redox potential) to enable DON to DOM-1 transformation [21]. Previously, similar findings were reported by Tally *et al*. [22], who found that anaerobes such as *Bacteroides oralis*, *Bacteroides melaninogenicus*, *Peptostreptococcus elsdenii* and *Fusobacterium nucleatum* were able to tolerate full oxygen exposure for 24 to 72 h. These observations suggest that the aforementioned bacteria are not strict anaerobes, and excessive oxygen exposure may inhibit their growth by inactivating key enzymes involved in anaerobic metabolism [23].

**Fig. 2.**
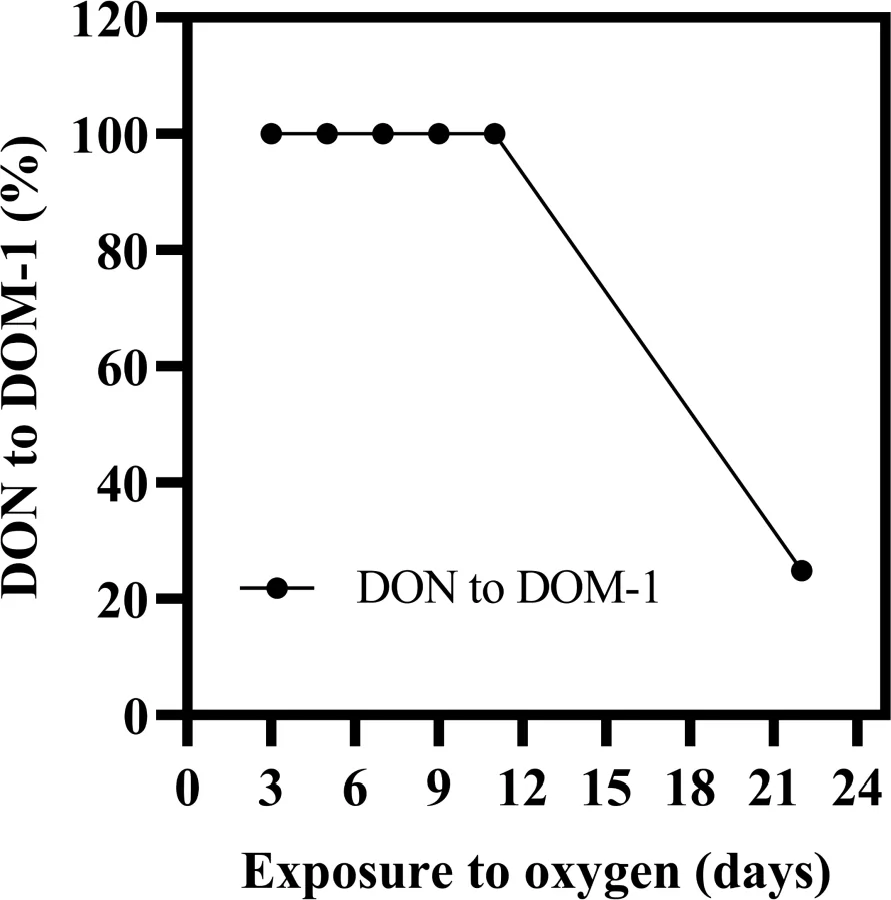
Detoxification activity (DON to DOM-1) of an initial bacterial culture grown in L10 broth (5 ml) after exposure to oxygen. The initial culture of *Bacillus* sp. LS100 was exposed to oxygen during incubation at 22 °C prior to the detoxification assay. *N*=3.

### Effect of culture scale-up and storage on detoxification activity

For practical applications, a large volume of the bacterial inoculum with desired functions and a long life-span during storage is normally required. To achieve this goal, *Bacillus* sp. LS100 was cultured in 300 ml of BHI broth and stored at 22 °C for up to 44 days. As shown in Fig. 3, the bacterium that was cultured, processed and stored under anaerobic conditions demonstrated full activity to detoxify DON after 35 days, but not after 44 days of storage. The full detoxification activity was also observed with the bacterium stored up to 30 days, which was cultured, processed and stored aerobically. These results suggest the possibility that *Bacillus* sp. LS100 can be scaled up to produce a large volume of the culture for inoculating animals such as pigs and stored for at least 1 month with the desired function to detoxify DON, which can facilitate the application of the bacterium in commercial swine production.

**Fig. 3.**
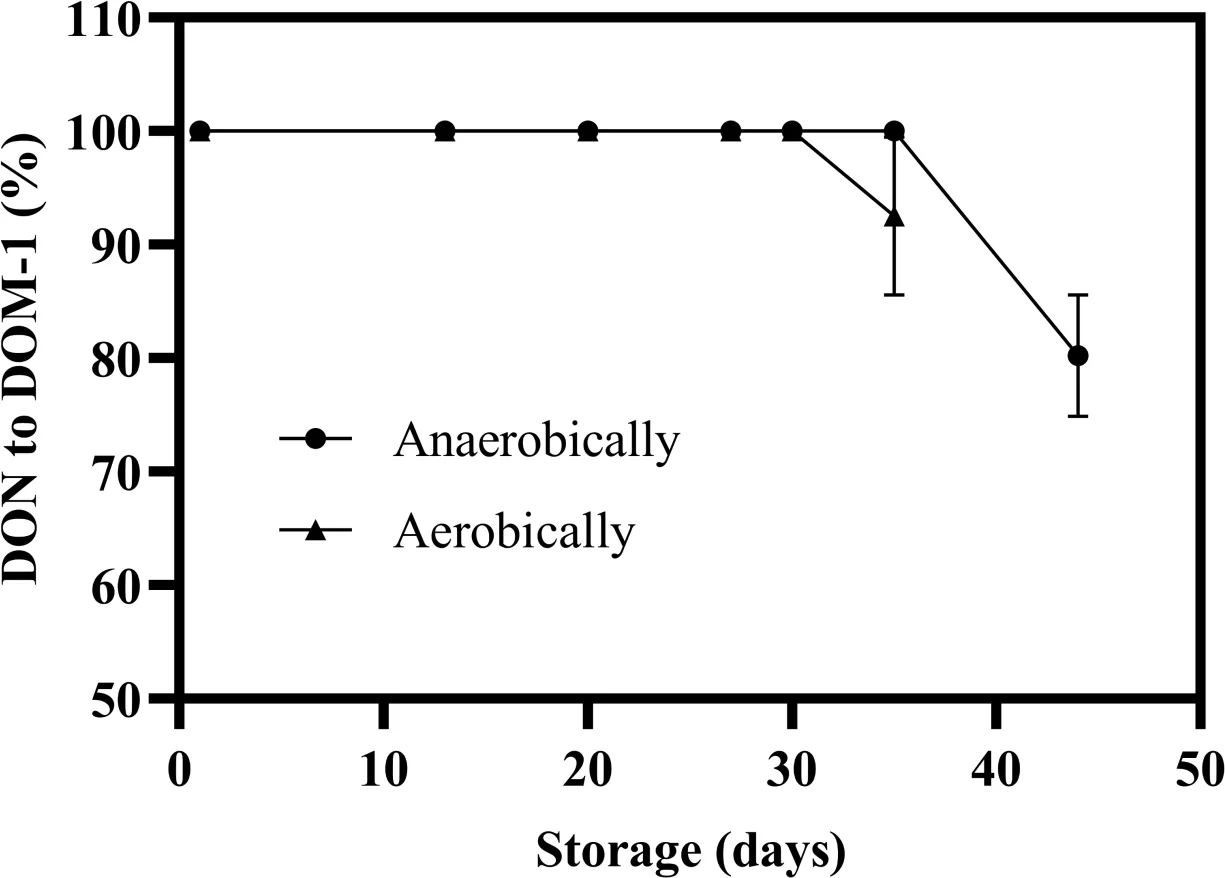
Detoxification activity (DON to DOM-1) of scaled-up bacterial cultures grown in BHI broth (300 ml) after storage at 22 °C. *N*=3.

### Detoxification of DON in mouldy corn

Table 3 exhibits the detoxification of DON by *Bacillus* sp. LS100 in PBS, L10, TSB and the mixtures of PBS and broth at different ratios after incubation for 24 and 48 h anaerobically. While the bacterium partially detoxified DON in PBS only, regardless of the incubation length, a full detoxification of DON was observed in L10 broth after 24 h incubation. In contrast to the results in L10, a full detoxification activity was achieved only after 48 h incubation in TSB broth. In the assay mixtures with different ratios of PBS to broth, the bacterium partially detoxified DON in TSB, regardless of the percentages of PBS after 24 h incubation. However, in L10 broth after 24 h, the bacterium achieved a full detoxification activity when the percentage of PBS in the assay mixtures was 50% and less. These results suggest the potential of the bacterium to remove DON from mouldy corn and provide a reference for reducing the cost in future applications. In parallel to detoxifying DON in mouldy corn under anaerobic conditions, the detoxification was also conducted aerobically. However, no detoxification activity of DON in mouldy corn was observed, further suggesting the requirement of an oxygen-free atmosphere by the bacterium to detoxify DON.

**Table 3.** DON breakdown (%) in mouldy corn by *Bacillus* LS100

Assay mixture	24 h incubation		48 h incubation	
	L10	TSB	L10	TSB
20% broth+80% PBS	89.5±12.1	75.4±34.7	99.0±1.45	100.0±0.00
30% broth+70% PBS	92.8±10.2	67.8±45.6	100.0±0.00	100.0±0.00
50% broth+50% PBS	100.0±0.00^a^	83.1±24.0	100.0±0.00	100.0±0.00
70% broth+30% PBS	100.0±0.00	86.5±19.0	100.0±0.00	100.0±0.00
100% broth+0% PBS	100.0±0.00	53.8±65.3	100.0±0.00	100.0±0.00

Note: 100% PBS served as a control with DON breakdown (%) of 71.2±43.7 and 96.6±3.71, respectively, after 24 and 48 h incubation. Additionally, 100% broth without an LS100 inoculum showed no detoxification activity of DON (0%) after 24 and 48 h incubation, respectively. *N*=3. The value labelled with ‘a’ in a column is significantly different from the control.

### Stability under mimicked gut conditions

*B. subtilis* DSM 32324 and *B. subtilis* DSM 32325 have been approved for use in the European Union countries as feed additives to detoxify DON [24]. These strains exhibited high stability under various environmental conditions, such as heat, gastric fluids and moisture [25]. To employ *Bacillus* sp. LS100 for *in vivo* detoxification of DON, it is essential to evaluate its stability in growth and DON-detoxifying activity under conditions similar to the animal gut environment, such as low pH, high bile salts and the presence of gastric or intestinal fluid. The sensitivity in growth of *Bacillus* sp. LS100 to low pH is presented in Fig. 4. The results indicated that *Bacillus* sp. LS100 achieved optimal growth in TSB broth at near-neutral pH. However, its growth declined significantly when the pH dropped to 5.0 and below. Consistent with our results, Cheng *et al*. [26] found that the DON removal ability of *B. subtilis* ZZ and *B. licheniformis* DY significantly decreased at pH values between 3.5 and 5. Similarly, Jia *et al*. [27] demonstrated that *B. subtilis* ASAG 216, isolated from the intestine of a donkey, showed higher DON degradation activity at pH values between 6.5 and 9.0. In contrast, a previous study reported that *B. licheniformis* YB9, isolated from mouldy soil, maintained DON degradation activity at pH values ranging from 2 to 4 for 48 h [28]. These discrepancies may be attributed to differences in pH sensitivity among *Bacillus* species or even among individual isolates, particularly in relation to their ability to form spores or produce specific enzymes [29].

**Fig. 4.**
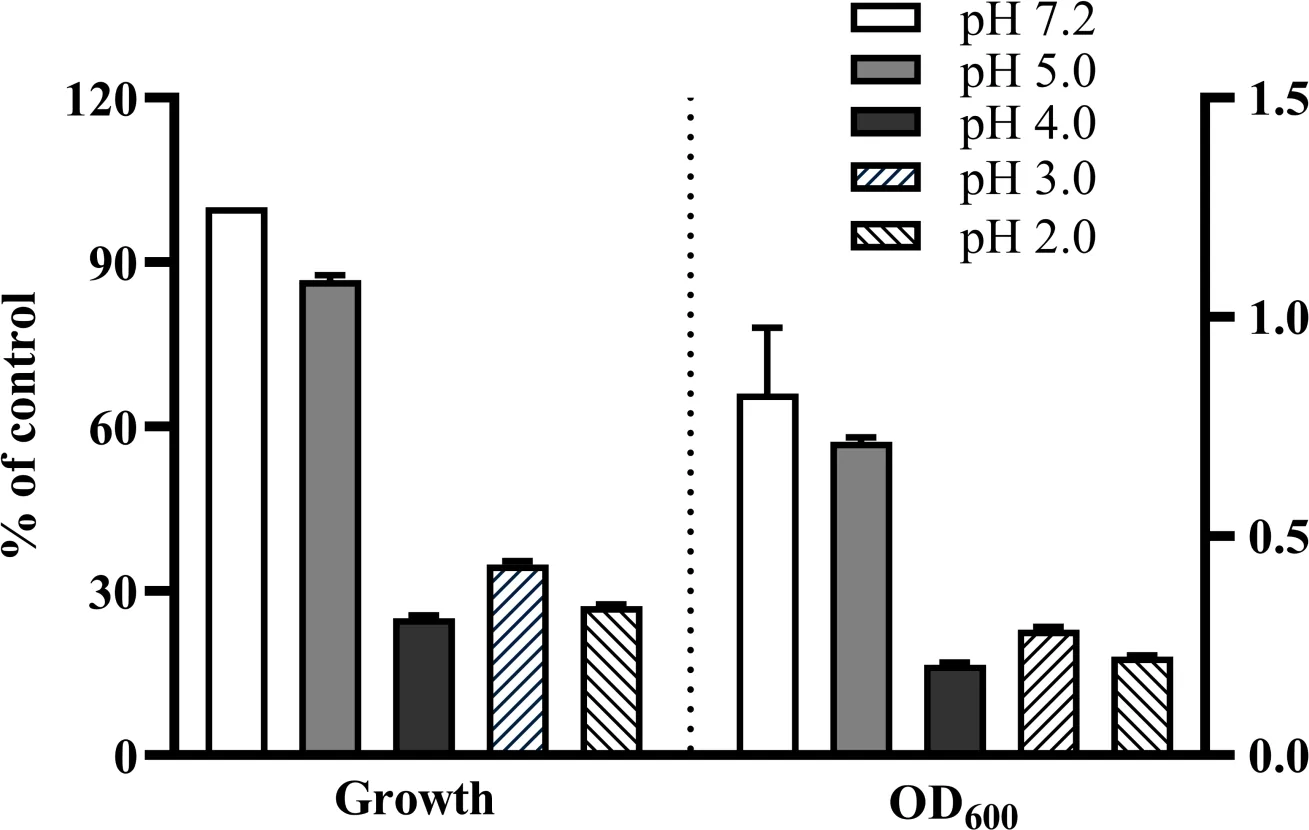
Effect of pretreatment at different pH values on the growth of *Bacillus* sp. LS100. The growth in TSB at pH 7.2 served as a control (100%). *N*=3.

The effect of pre-treatment with pig SF on the growth and DON-detoxifying activity of *Bacillus* sp. LS100 was also examined, since stomach acidity, primarily due to hydrochloric acid secretion, plays a key role in food/feed digestion and pathogen defence in animals [30]. As shown in Table 4, the growth of *Bacillus* LS100 in L10 broth was significantly reduced following treatment with pig SF for 1, 2 and 3 h. This treatment also abolished the bacterium’s ability to detoxify DON. In contrast, a previous study reported that isolates of different *Bacillus* species, including *B. subtilis*, *B. pumilus*, *B. licheniformis*, *B. clausii*, *B. megaterium*, *B. firmus* and *B. cereus*, from the intestines of broilers were able to tolerate gastrointestinal tract conditions, including the acidic SF [31]. Recently, Golnari *et al*. [32] also described that isolates from eight different *Bacillus* spp. with different origins showed a high resistance to gastrointestinal tract conditions. These observations further highlight the substantial diversity among *Bacillus* species, particularly in their functional capabilities and adaptability to various environmental conditions.

**Table 4.** DON removal by *Bacillus* sp. LS100 pretreated with pig SF

Pretreatment	Initial culture (24 h)		Initial culture (48 h)		Subculture (24 h)	
	DON conversion (%)	Growth (%)	DON conversion (%)	Growth (%)	DON conversion (%)	Growth (%)
PBS (control)	100±0.00^a^	100.0±0.00^a^	100±0.00^a^	100.0±0.00^a^	100±0.00^a^	100.0±0.00^a^
SF 1 h	0±0.00^b^	23.3±2.99^b^	0±0.00^b^	58.4±16.94^b^	0±0.00^b^	67.0±15.50^b^
SF 2 h	0±0.00	22.0±0.68^b^	0±0.00	54.3±5.44^b^	0±0.00	53.5±34.74^b^
SF 3 h	0±0.00	21.6±1.52^b^	0±0.00	62.1±5.18^b^	0±0.00	51.4±1.57^b^

Note: The pretreatment with SF was carried out for up to 3 h followed by incubation in L10 broth for measuring bacterial growth and detoxification activity. The subculture was developed from the 48 h-grown culture. *N*=3. The values labelled with different letters in a column are significantly different (*P*≤0.05).

Bile tolerance is important for the successful colonization of the small intestine by ingested bacteria [33]. The bile salt sensitivity of *Bacillus* sp. LS100 grown in TSB broth is exhibited in Fig. 5. The results demonstrated that very little growth of *Bacillus* sp. LS100 was observed within the tested range of bile salts, suggesting that *Bacillus* sp. LS100 was sensitive to bile salts at a concentration of 0.1% and above. Similar to our findings, Kristoffersen *et al*. [34] reported that *B. cereus* strains isolated from 40 different sources exhibited low tolerance to bile salts, even at a concentration of 0.01%. However, Guo *et al*. [35] found that *B. subtilis* MA139 showed high bile salt tolerance, remaining viable even at a concentration of 0.3%. The differences in bile salt tolerance may have been attributed to variations in the cell wall and membrane compositions among *Bacillus* strains, which affect their ability to resist bile salt-induced damage [36]. *Bacillus* sp. LS100 appeared to be sensitive to bile salts above 0.1% in the present study. Given that LS100 was originally isolated from the chicken intestine and *Bacillus* bacteria are often found in the gastrointestinal tracts of different animals, isolate LS100 could have the potential to survive in the pig intestine. Previous research has shown that *B. subtilis* could improve the survivability of lactic acid bacteria during food preparation, storage and ingestion [37]. This may suggest that the coexistence of *Bacillus* sp. LS100 with lactic acid bacteria in the pig intestine may increase the survival potential of LS100. *Bacillus* group are spore-forming bacteria, and their spores are resistant to various environmental stresses. Thus, there is an opportunity to use LS100 as the spore form in application to increase its resistance to high bile salts, if the bacterium can be induced to produce spores. In addition, target-release encapsulation strategies could be developed to enable efficient release at intestinal sites of DON absorption, thereby improving detoxification efficacy.

**Fig. 5.**
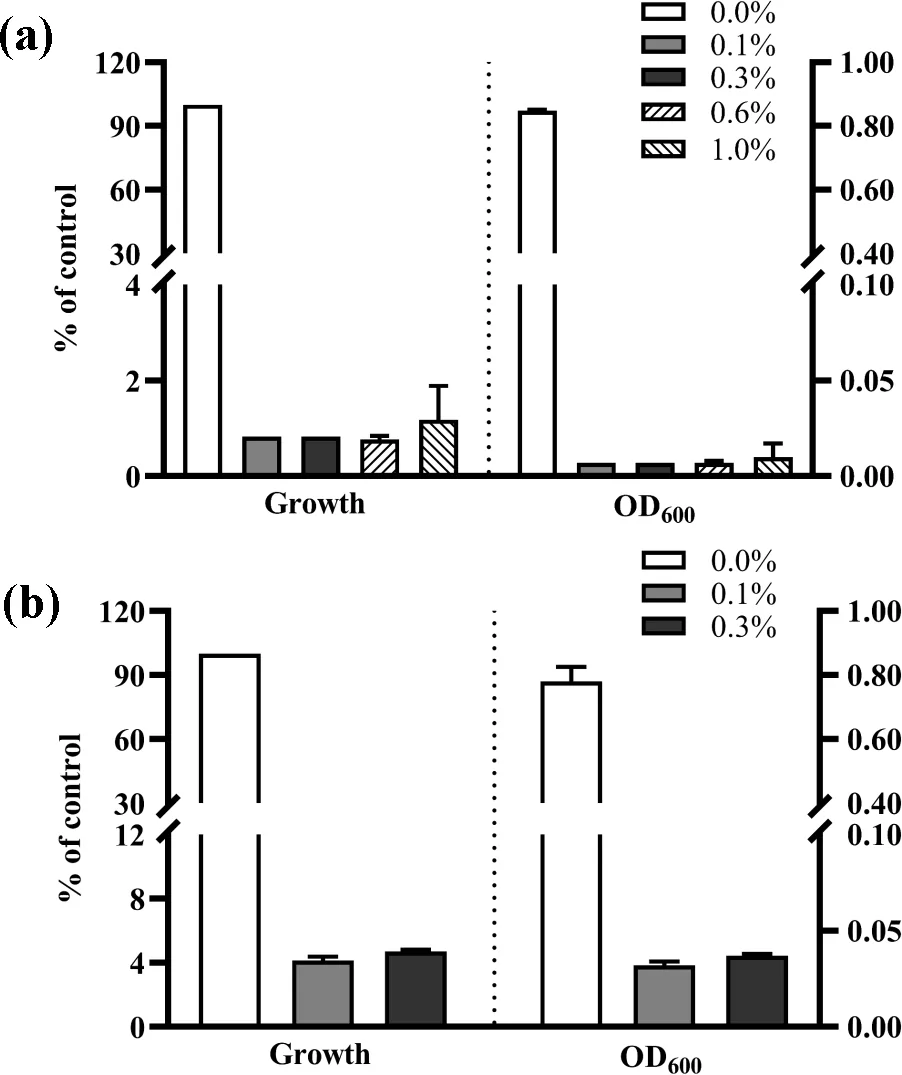
Growth of *Bacillus* sp. LS100 in the presence of different concentrations of bile salts. (**a**) 1% inoculum; (**b**) 10% inoculum. The growth in TSB without any supplementation of bile salts served as a control (100%).

The jejunum and ileum are the segments of the small intestine and are primarily responsible for completing chemical digestion and absorbing most nutrients from ingested feed/food in animals [38]. It is crucial to assess whether *Bacillus* sp. LS100 can exert its function in the jejunum and ileum, as well as determine the timing of its activity within these intestinal segments. In the present study, as illustrated in Table 5, the ability for *Bacillus* sp. LS100 to detoxify DON did not retain well in PBS containing the digesta without L10 broth. However, *Bacillus* sp. LS100 retained a full or near-full DON breakdown activity in the presence of either jejunum or ileum digesta after 24 or 48 h of anaerobic incubation in L10 broth. Complete detoxification of DON was also observed after 24 and 48 h incubation in the presence of ileum digesta, even with 50% PBS and 50% L10 broth. When the JED was suspended in the mixture of PBS and L10 broth, longer incubation was required for *Bacillus* sp. LS100 to exert its activity to detoxify DON compared with the ileum digesta. Based on all the results described above, protection of *Bacillus* sp. LS100 by encapsulation was considered necessary to ensure its effective delivery to the animal intestines and to achieve full detoxification efficacy in animals.

**Table 5.** DON removal by *Bacillus* sp. LS100 in the presence of the pig JED or ILD

Assay mixture	12 h incubation DON conversion (%)		24 h incubation DON conversion (%)		48 h incubation DON conversion (%)	
	JED	ILD	JED	ILD	JED	ILD
PBS	0±0.00	6.0±11.98^b^	0±0.00^b^	50.0±57.74^b^	1.1±2.24^c^	50.0±57.74^b^
50% L10+50% PBS	0±0.00	77.0±36.37^a^	37.4±43.99^b^	100.0±0.00^a^	66.1±47.16^b^	100.0±0.00^a^
L10	13.6±14.90	78.8±25.27^a^	95.9±8.25^a^	100.0±0.00^a^	100.0±0.00^a^	100.0±0.00^a^

Note: All the assay mixtures contained ~10% digesta in L10 broth. *N*=3. The values labelled with different letters in a column are significantly different (*P*≤0.05).

### Effect of encapsulation

Encapsulation is an effective strategy to protect cells by embedding them within a carrier matrix [39]. There is an increasing need for encapsulated micro-organisms to enhance their efficacy, as many are unstable under the harsh conditions of the digestive tract in animals [40–42]. In the present study, encapsulation of *Bacillus* sp. LS100 was tested with regard to retaining its activity to detoxify DON. The beads (wet and dry) of encapsulated *Bacillus* sp. LS100 had the size of ~2.0 mm in diameter. HPLC analysis was employed to determine the conversion of DON to DOM-1. In the HPLC profile, the characteristic peak for DON appeared at a retention time of ~3.76 min, whereas the fingerprint peak of DOM-1 was at ~5.1 min, respectively (Fig. 6a). Empty beads (without LS100) were also evaluated to determine if the bead matrix itself could degrade or bind DON. The result showed only the signature DON peak in the assay mixtures. Furthermore, an equivalent concentration of DON was recovered from the samples after removal of the beads, indicating that the empty beads did not affect DON conversion to DOM-1 by either degrading or absorbing DON under the current experimental conditions. The activity of encapsulated *Bacillus* sp. LS100 to convert DON to DOM-1 was examined after 8, 10 and 14 days of storage. While the encapsulated bacterium in the form of dry beads lost its activity to convert DON to DOM-1 after 10 days of storage at 22 °C, the same bacterium preparation retained its activity at 8 days of storage. Differently, the bacterium in the wet beads retained its detoxification activity at 8, 10 and 14 days of storage at 22 °C (Fig. 6b, only the 10 days data presented). The bacterium in the wet beads after 14 days of storage was subsequently treated in SGF for 2 h, and a significant level of detoxifying activity was still detected (Fig. 6c). In contrast, SGF treatment abolished the detoxification activity of the unencapsulated bacterium. These findings suggest that encapsulation technology could protect *Bacillus* sp. LS100 from deactivation under harsh gastric conditions. When ingested through feed, DON primarily damages the small intestine of pigs, with the jejunum being the most sensitive site, followed by the ileum and duodenum [43, 44]. *Bacillus* sp. LS100 exhibits minimal growth at pH 4 and below (Fig. 2, Table 2) but grows well at pH values above 5, up to pH 7.2. It also retains detoxification activity in ILD, which has a pH of 6.0–6.8 [45]. Although *Bacillus* sp. LS100 demonstrated stronger DON-detoxification activity in ILD than in JED (Table 3), the jejunum remains a likely optimal site for its release from microencapsulation beads. These findings provide a foundation for further optimizing the encapsulation strategy to improve the DON-detoxification efficacy of this bacterium in swine.

**Fig. 6.**
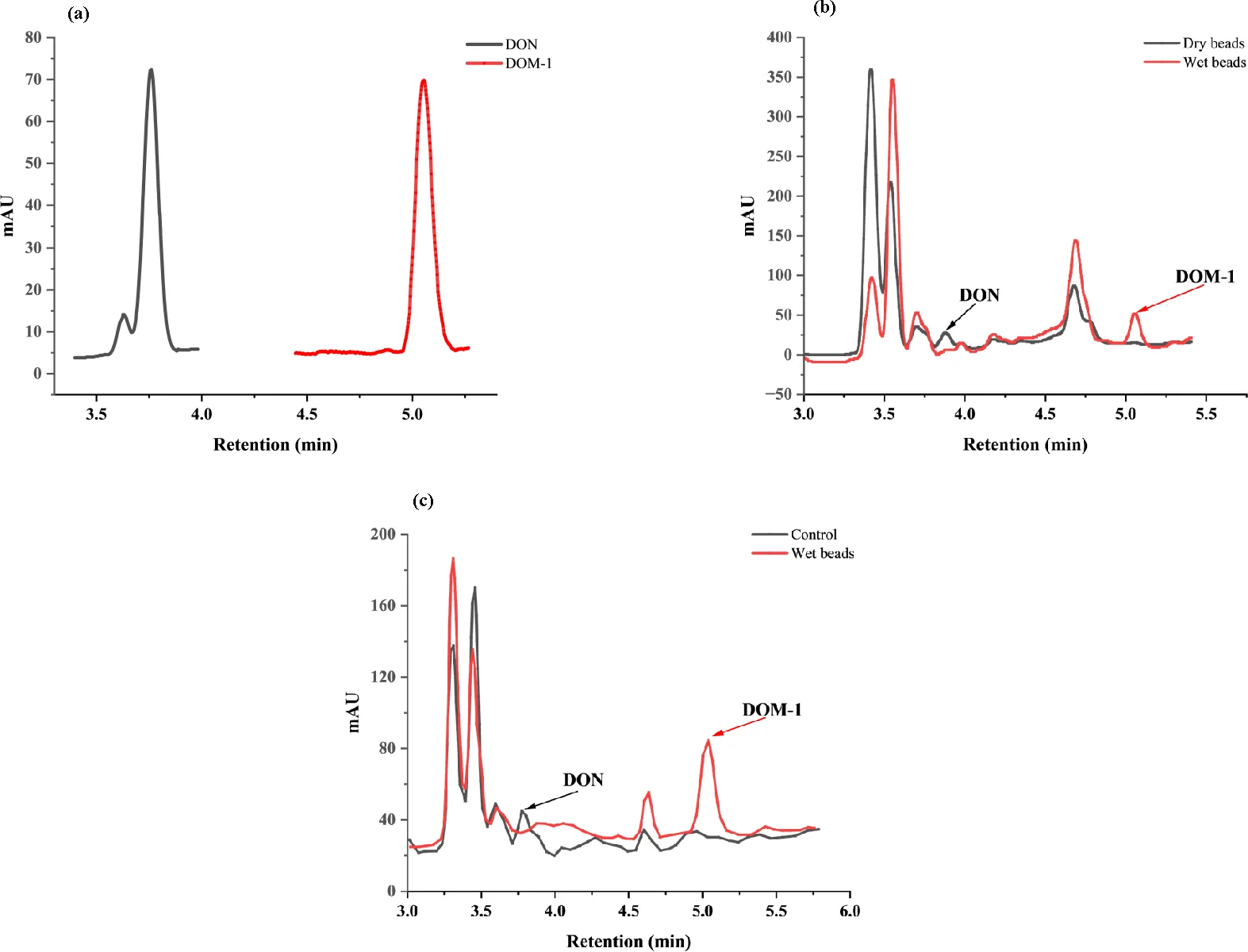
Signatures of the HPLC spectrum of DON and DOM-1 showing the transformation from DON to DOM-1 by encapsulated *Bacillus* LS100. (**a**) DON (40 p.p.m.) with retention time at ~3.76 min and DOM-1 with retention time at ~5.10 min; (**b**) Transformation of DON to DOM-1 by encapsulated *Bacillus* LS100: red line – wet beads; black line – dry beads; (**c**) transformation of DON to DOM-1 by encapsulated *Bacillus* LS100 in the wet beads after 14 days of storage at 22 °C followed by treatment in SGF at 22 °C for 2 h. Red line – wet beads; black line – unencapsulated bacterium (control). The L10 broth for the assay contained 10 p.p.m. DON.

## Conclusion

This research demonstrates that the bacterial isolate *Bacillus* sp. LS100 has the potential to be used in feed as an additive to detoxify DON in the pig gut. Microencapsulation is necessary to protect the bacterium during gastrointestinal transit and to ensure its effective delivery and functionality in pigs.

## Abbreviations

AIM: anaerobic incubation medium
BHI: brain heart infusion
CecExt: caecal content extract
DOM-1: de-epoxy deoxynivalenol
DON: deoxynivalenol
ILD: ileal digesta
JED: jejunal digesta
LC-MS: liquid chromatography–MS
LOD: limit of detection
LOQ: limit of quantification
SF: stomach fluid
SGF: simulated gastric fluid
TSB: tryptic soy broth
TSB: tryptic soy broth
